# Differential Expression of Tissue Transglutaminase Splice Variants in Peripheral Blood Mononuclear Cells of Primary Progressive Multiple Sclerosis Patients

**DOI:** 10.3390/medsci6040108

**Published:** 2018-11-27

**Authors:** Claudia Sestito, John J. P. Brevé, Joep Killestein, Charlotte E. Teunissen, Micha M. M. Wilhelmus, Benjamin Drukarch, Anne-Marie van Dam

**Affiliations:** 1 Department of Anatomy and Neurosciences, Amsterdam UMC, Vrije Universiteit Amsterdam, 1081 HZ Amsterdam, The Netherlands; c.sestito@vumc.nl (C.S.); jjp.breve@vumc.nl (J.J.P.B.); m.wilhelmus@vumc.nl (M.M.M.W.); b.drukarch@vumc.nl (B.D.); 2 Department of Neurology, Amsterdam UMC, Vrije Universiteit Amsterdam, 1081 HZ Amsterdam, The Netherlands; j.killestein@vumc.nl; 3 Department of Clinical Chemistry, Amsterdam UMC, Vrije Universiteit Amsterdam, 1081 HZ Amsterdam, The Netherlands; ce.teunissen@vumc.nl

**Keywords:** Tissue transglutaminase, alternative splicing, multiple sclerosis, peripheral blood mononuclear cells (PBMC)

## Abstract

Multiple Sclerosis (MS) is an inflammatory and neurodegenerative disorder of the central nervous system (CNS) characterized by inflammation and immune cell infiltration in the brain parenchyma. Tissue transglutaminase (TG2), a calcium-dependent cross-linking enzyme, has been shown to be present in infiltrating MHC-II positive cells in lesions of patients suffering from MS. Moreover, TG2 mRNA levels in peripheral blood mononuclear cells (PBMC)-derived from primary progressive (PP)-MS patients correlated with clinical parameters, thus highlighting the importance of TG2 in MS pathology. In the present study, we further characterized TG2 expression by measuring the mRNA levels of full-length TG2 and four TG2 alternative splice variants in PBMCs derived from PP-MS patients and healthy control (HC) subjects. In PP-MS-derived PBMCs, TG2 variant V4b was significantly higher expressed, and both V4a and V4b variants were relatively more expressed in relation to full-length TG2. These observations open new avenues to unravel the importance of TG2 alternative splicing in the pathophysiology of PP-MS.

## 1. Introduction

Multiple Sclerosis (MS) is a chronic inflammatory and degenerative disorder of the central nervous system (CNS). Clinically, it can be divided into various subtypes including relapsing-remitting MS (RR-MS) which results in episodes of neurological dysfunction followed by remission and primary progressive MS (PP-MS) characterized by progressive neurological dysfunction from the onset of the disease without remission phases [1]. Pathologically, MS is characterized by immune cell infiltration into the CNS, inflammation, demyelination, and ultimately axonal loss [2]. Peripheral blood mononuclear cells (PBMCs), consisting of lymphocytes and monocytes among others, represent the main infiltrating cells. These immune cells, and the subsequent inflammatory responses, have been shown to play important roles in the onset and progress of MS pathology [3]. 

Tissue Transglutaminase (TG2) is a 78 kDa enzyme that is ubiquitously expressed. Its enzymatic function as a Ca^2+^-mediated crosslinking enzyme is best characterized, but it can also bind and hydrolyze GTP/ATP. It has extensively been demonstrated that Ca^2+^ and GTP/GDP binding inversely regulate the transamidating activity of TG2 by inducing a conformational change; indeed, TG2 is active as a Transglutaminase (TGase) when bound to Ca^2+^ and in an open conformation. On the contrary, it is enzymatically inactive when bound to GTP/GDP and is then maintained in a closed conformation [4]. The expression and/or enzymatic activity of TG2 are enhanced by inflammatory mediators, as is evident by the presence of inflammation-related response elements in the TG2 promotor region [5,6,7]. In addition to the full-length (V1) TG2 mRNA, four alternative splice variants of TG2 mRNA have been described, named V2, V3, V4a, and V4b [8], all producing shorter TG2 isoforms than the V1 full-length TG2, with predicted different (enzymatic) properties/functions. The common feature of all short variants is that they lose, to a certain extent, their C-terminus, which contains the GTP binding domain. The loss of amino acids at the C-terminus reduces GTP binding to the enzyme, thereby allowing the short variants to escape the GTP regulation. This results in TGase activity when there is a transient increase in Ca^2+^ levels [9]. The V2 variant (TGM2_v2, 62 kDa) was observed to be increased in post-mortem brain tissue of Alzheimer’s disease patients suggesting a role in Alzheimer’s disease pathology [10]. Interestingly, this variant has also been described to have opposite effects on cells as it was found to induce cell differentiation in neuroblastoma cells but cell death in NIH3T3 fibroblasts, unlike the full-length TG2 which acted as repressor of differentiation in neuroblastoma cells and growth-inducer in NIH3T3 cells [11,12]. V3 (TGM2_V3, 38 kDa) mRNA was found to be significantly increased in leukocytes of coeliac disease patients compared to healthy control (HC) subjects, thus suggesting a role in this disease [13]. V4a and V4b mRNA (TGM2_V4a and TGM2_V4b, 75 kDa and 70 kDa, respectively) are predominantly expressed in leukocytes [14] but their function is unknown. Recently, we demonstrated that total TG2 mRNA levels were increased in monocytes derived from MS patients compared to HC subjects [15]. In addition, we observed that the expression of total TG2 in PBMCs was altered in different subtypes of MS patients, with PP-MS patient-derived PBMCs expressing the most TG2 mRNA. Moreover, in PP-MS patients, TG2 mRNA levels correlated with various clinical parameters [16], suggesting that TG2 expression in circulating PBMCs derived from PP-MS patients may be of clinical relevance. To further characterize TG2 expression in PP-MS patients, in this study we investigated the expression profile of full-length TG2 and TG2 splice variants in PBMCs of PP-MS patients compared to those of HC subjects. This may give insight in the possible contribution of TG2 isoforms to MS pathophysiology, and in particular to that of PP-MS patients.

## 2. Materials and Methods

### 2.1. Subjects

From the MS Center Amsterdam (VU University Medical Center), a total of 22 MS patients were included in the present study. The patients were diagnosed with MS according to the 2005 Revisions to the McDonald Diagnostic Criteria [17] and classified as PP-MS patients [1]. In addition, a total of 30 age and gender-matched HC volunteers were included. The study was approved by the local ethical committee (protocol number 04.009). Written informed consent was obtained from all participants. In the PP-MS group, 9.1% of the patients (*n* = 2) were treated with disease modifying treatments (DMT, *n* = 1 interferon β; *n* = 1 methotrexate) (Table 1).

### 2.2. Isolation of Primary Human Peripheral Blood Mononuclear Cells

At the baseline visit, peripheral blood was drawn by venipuncture, collected into ethylenediaminetetraacetic acid (EDTA) tubes (Becton Dickinson), centrifuged for 10 min at 1500× *g* at 4 °C and stored at −80 °C. PBMCs were isolated from 22 MS patients and 30 HC by density centrifugation using Ficoll (Ficoll Isopaque PLUS, GE Healthcare, Uppsala, Sweden) according to the manufacturer’s instructions and stored in liquid nitrogen until further processing. 

### 2.3. mRNA Isolation and cDNA Synthesis

Total RNA was isolated from primary human PBMCs using TRIzol Reagent (Invitrogen, Carlsbad, CA, USA) according to the manufacturer’s instructions. Total RNA was further purified using the MicroElute RNA clean up kit (Omega bio-tek, Norcross, GA, USA). The RNA purity was assessed using the NanoDrop 1000 (Thermo Fisher Scientific, Waltham, MA, USA). The quality ratios 260/280 and 260/230 were above 1.9 and 1.7, respectively for all the samples included in the study. Total RNA (200 ng/sample) was reverse-transcribed into cDNA using the High-Capacity cDNA Reverse Transcription kit (Applied Biosystems, Foster City, CA, USA) according to the manufacturer’s instructions. 

### 2.4. Semi-Quantitative Real-Time PCR (qPCR)

For qPCR, the Power SYBR Green Master Mix (Life Technologies, Carlsbad, CA, USA) was used. Primers sequences for detecting full length (V1), and V3, V4a and V4b TG2 splice variants were obtained from Phatak et al. [8]. An intron spanning V2 primer pair was newly developed to prevent genomic DNA amplification. Primers were purchased from Eurogentec (Liège, Belgium) and qPCR was performed in MicroAmp Optical 96-well Reaction Plates (Applied Biosystems) on a StepOnePlus Real-Time PCR system (Applied Biosystems). The reaction mixture with a total volume of 10 μL was composed of 1× Power SYBR Green buffer (Applied Biosystems), 1.88 pmol of each primer (Table 2) and 5 ng cDNA. The thermal cycling conditions were an initial 10 min at 95 °C followed by 50 cycles of 15 s at 95 °C and 1 min at 60 °C. The specificity of the reaction was checked by melt curve analysis of the individual qPCR reactions. The relative expression level of the target genes was determined by the LinRegPCR software (http://www.hfrc.nl, downloads, applications, linreg PCR, version 2014.3) using the following calculation N0 = Nq/ECq (N0 = target quantity, Nq = fluorescence threshold value, E = mean PCR efficiency per amplicon, Cq = threshold cycle [18], after which the value was normalized relative to the geometric mean of the mRNA levels hypoxanthine phosphoribosyltransferase 1(HPRT1) and polymerase (RNA) II polypeptide F (POLR2F). HPRT1 and POLR2F were chosen as reference genes based on the results of the GeNorm software analysis (version 3.5) in which the stability of six different human housekeeping genes (GAPDH, MRIP, POLR2F, HPRT1, PGK1, SDHA) was assessed.

### 2.5. Statistical Analysis

Because the mRNA level of TG2 splice variants was undetectable in several samples, sample size differs between the groups. Based on unreliable qPCR outcome (extreme values) one HC sample was excluded from V3 dataset and one MS patient sample from the V4a dataset. For the two group analysis on the expression level of each TG2 variant, data were analyzed using the nonparametric test Mann–Whitney U, because of non-normal distribution of the data. For the two group analysis on the percentage of TG2 splice variant in relation to V1 expression, an independent Student *t*-test was performed on V2/V1 and V4a/V1 while for V3/V1 and V4b/V1 an independent Student *t*-test was performed on the logarithmic transformed ratios, because of non-normal distribution of the data. *p* < 0.05 was considered statistically significant. All the analyses were performed using SPSS version 22.0 (IBM Corp, Armonk, NY, USA).

## 3. Results

### Differential Expression of TG2 Splice Variants in PP-MS Patients Compared to HC Subjects

**Figure 1:**
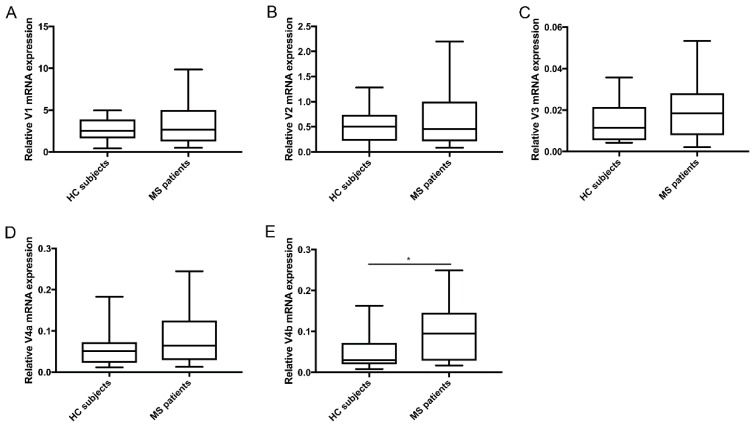
mRNA levels of TG2 isoforms in PBMCs derived from PP-MS patients and HC subjects. qPCR analysis was performed to detect (**A**) V1 mRNA (HC: N = 30/PP-MS: N = 22); (**B**) V2 mRNA (HC: N = 30/PP-MS: N = 22); (**C**) V3 mRNA (HC: N = 18/PP-MS: N = 12); (**D**) V4a mRNA (HC: N = 24/PP-MS: N = 20) and (**E**) V4b mRNA (HC: N = 23/PP-MS: N = 18) in primary human peripheral blood mononuclear cells (PBMCs) isolated from PP-MS patients and HC subjects. Data are normalized relative to the geometric mean of the mRNA levels HPRT1 and POLR2F. mRNA and are shown in box-and-whiskers plots in which the median is represented by a horizontal line within the box and the lower and upper whiskers represent the min and max value. * *p* < 0.05. MS = Multiple sclerosis, HC = Healthy control.

First, we determined the expression levels of full-length TG2 (V1) and the splice variants (V2, V3, V4a, and V4b) in PBMCs of PP-MS patients and HC subjects (Figure 1A–E). All TG2 variants were expressed by PBMCs from HC subjects and PP-MS patients, although at various levels. V1 was the highest expressed both in HC subjects and in PP-MS patients (median (min-max): HC: 2.53 (0.42–4.97); MS: 2.64 (0.47–9.95)), followed by V2 (median (min–max): HC: 0.50 (0.0008–1.28); MS: 0.45 (0.08–2.3)). The splice variants V4a and 4b were relatively equally expressed (median (min–max): HC: 0.051 (0.011–0.18); MS: 0.064 (0.013–0.24) and HC: 0.03 (0.008–0.16); MS: 0.095 (0.017–0.25), respectively) but undetectable in some samples (HC: *n* = 6 and *n* = 7; PP-MS: *n* = 1 and *n* = 4, respectively). The V3 splice variant had the lowest expression (median (min–max): HC: 0.011 (0.004–0.036); MS: 0.018 (0.002–0.053)) and was undetectable in a significant number of HC subjects (*n* = 11) and PP-MS patients (*n* = 10), suggesting that this isoform is not highly expressed by PBMCs. When comparing expression levels between HC subjects and PP-MS patients, the mRNA levels of the short variant V4b were significantly higher in PP-MS patients than in HC subjects (*p* = 0.03) (Figure 1E). 

**Figure 2:**
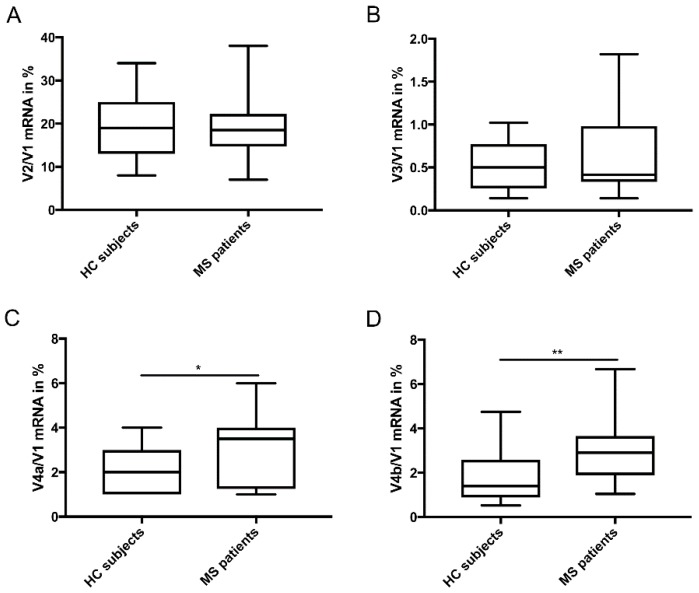
mRNA levels of TG2 short isoforms as percentage of V1 full length mRNA. (**A**) Percentage of V2 expression over V1 mRNA (HC: N = 30/PP-MS: N = 22); (**B**) Percentage of V3 expression over V1 mRNA (HC: N = 18/PP-MS: N = 12); (**C**) Percentage of V4a expression over V1 mRNA (HC: N = 24/PP-MS: N = 20); (**D**) Percentage of V4b expression over V1 mRNA (HC: N = 23/MS: N = 18) in PBMCs isolated from PP-MS patients and HC subjects. Data are presented as percentage of V1 (full-length) expression. mRNA data are normalized relative to the geometric mean of the mRNA levels HPRT1 and POLR2F. mRNA data are shown in box-and-whiskers plots in which the median is represented by a horizontal line within the box and the lower and upper whiskers represent the min and max value. * *p* < 0.05 ** *p* < 0.01. MS = Multiple sclerosis, HC = Healthy control.

Furthermore, analysis of splice variant expression in relation to full-length V1 mRNA (Figure 2A–D) showed that the percentage of V4a and V4b expression in relation to V1 mRNA was significantly higher in PP-MS patients compared to HC subjects (*p* = 0.013 and *p*= 0.003, respectively) (Figure 2C,D).

## 4. Discussion

TG2 is a pleiotropic enzyme that exerts several enzymatic and non-enzymatic functions [4]. As many cellular proteins are potential TG2 substrates, its enzymatic activity is strictly regulated by GTP/GDP and Ca^2+^ availability. By displaying an alternative C-terminus splicing (contains the GTP binding domain), the four splice variants of TG2 can escape physiological regulation of its transamidation activity [9]. The expression levels of these variants are found to be altered in several diseases [10,13] which might indicate that under pathological conditions, their role could be due, at least partly, to a relative low intracellular Ca^2+^ concentration needed for their activation. In PP-MS, we recently observed that total TG2 expression in PBMCs is relatively high. In the present study, we analyzed TG2 splice variant expression by these cells in PP-MS patients versus HC subjects. In general, the TG2 splice variants mRNA levels measured in PBMCs were lower as compared to those measured in various cancer cell lines [8]. Still, full-length TG2 (V1) was the highest expressed, which is in line with what has been shown before in polymorphonuclear cells, lymphocytes, monocytes and other cell lines [8,13]. The splice variants V4a and V4b were expressed in PBMCs but their expression level was lower than that of full-length TG2 mRNA which is different from what has been shown before in leucocytes [13,14]. 

The observation that V4b mRNA levels and the percentage of V4a and V4b in relation to full-length V1 TG2 were significantly higher in PP-MS patients compared to HC subjects suggest that during the disease, an increased alternative splicing of the full-length TG2 gene occurs with a preference for V4a and V4b mRNA generation. This profile observed in PP-MS patient-derived PBMCs might represent a TG2-specific signature that could be of clinical relevance as a diagnostic biomarker for PP-MS patients. In addition, by exhibiting TGase activity when a transient increase in Ca^2+^ levels occurs, the splice variants could be directly implicated in the underlying pathological process by inducing e.g., the activation of phospholipase A_2_ [19], known to play a pivotal role in inflammation and MS [20]. Clearly, further clinical and pathological studies are needed to elucidate these potential implications. For instance, PBMCs actually are a heterogeneous population of immune cells and future studies should aim to identify the main cell type expressing the splice variants. Subsequent longitudinal expression studies in PP-MS patients may further reveal the specificity of TG2 variant expression as markers for the disease or its progression. Nevertheless, because of TG2 involvement in other neurodegenerative diseases (e.g., Alzheimer’s disease [21,22]), further research is also needed to address whether the observed increase in relative V4a and V4b expression is restricted to MS pathology or if it is a common feature during neurodegenerative processes. 

In cancer, large-scale alterations in alternative splicing have been reported and it has been postulated that in cancer cells, alternative splicing is a more active process adopted by the cells to produce proteins that promote growth and survival. Interestingly, it has been demonstrated that there is a preferential expression of TG2 splice variants during cancer [8]. Also, during immune cell activation alternative splicing occurs, in particular about 50% of the genes expressed are alternatively spliced during T-cell activation [23]. In addition, alternative splicing is also frequently reported in (experimental) MS, where it has been shown that genes expressed by immune cells often undergo alternative splicing [24,25]. Therefore, similarly to what is suggested in cancer, alternative splicing could represent an active process occurring during inflammation in MS and TG2 mRNA expression could be directly affected by alternative splicing during immune cell activation.

Taken together, our data are the first to report expression of TG2 splice variants in MS patients. We demonstrated that in PBMCs derived from PP-MS patients there is a preferential expression of V4a and V4b TG2 splice variants in relation to full-length V1. Based on our observations, it is tempting to speculate that, by displaying dysregulated activity and subsequent functions (e.g., altered cross-linking activity and interaction with different protein), TG2 splice variants may participate in the underlying pathological mechanisms in PP-MS patients. We conclude that our observations open new avenues to unravel the importance of TG2 alternative splicing in the pathophysiology of PP-MS. 

## Figures and Tables

**Table 1:** Patient information.

Subjects	Number	Female/Male	Age (years) ± SD	Disease Duration (years) ± SD
HC	30	10/20	51.4 ± 3.3	NA
PP-MS	22	8/14	51.5 ± 7.9	9.1 ± 5.5

HC = healthy control; PP-MS = primary progressive MS; SD = standard deviation; NA = not applicable.

**Table 2:** Primer sequences.

Gene	Forward	Reverse
TGM2_v1 (full-length) *	5′ CCTTACGGAGTCCAACCTCA 3′	5′ CCGTCTTCTGCTCCTCAGTC 3′
TGM2_v2	5′ TACCCAGAGGGGTCCTCAGA 3′	5′ GGAACACAGGGCTTTACCAGA 3′
TGM2_v3 *	5′ GGTGAGTGGCATGGTCAACT 3′	5′ AGGGCTCATGACCCACATC 3′
TGM2_v4a *	5′ CCTTACGGAGTCCAACCTCA 3′	5′ CTGGGATGTGGAGGTGCA 3′
TGM2_v4b *	5′ CCTTACGGAGTCCAACCTCA 3′	5′ CACTGGTGTGGAGGTGCAGC 3′
POLR2F	5′ GAACTCAAGGCCCGAAAG 3′	5′ TGATGATGAGCTCGTCCAC 3′
HPRT1	5′ AGCCCTGGCGTCGTGATTAGT 3′	5′ CGAGCAAGACGTTCAGTCCTGTCC 3′

TGM2 = Transglutaminase 2; POLR2F = polymerase (RNA) II polypeptide F; HPRT1 = hypoxanthine phosphoribosyltransferase 1; * primer sequences were obtained from [8].
